# Early Adoption of Anti–SARS-CoV-2 Pharmacotherapies Among US Veterans With Mild to Moderate COVID-19, January and February 2022

**DOI:** 10.1001/jamanetworkopen.2022.41434

**Published:** 2022-11-11

**Authors:** Kristina L. Bajema, Xiao Qing Wang, Denise M. Hynes, Mazhgan Rowneki, Alex Hickok, Francesca Cunningham, Amy Bohnert, Edward J. Boyko, Theodore J. Iwashyna, Matthew L. Maciejewski, Elizabeth M. Viglianti, Elani Streja, Lei Yan, Mihaela Aslan, Grant D. Huang, George N. Ioannou

**Affiliations:** 1 Veterans Affairs Portland Health Care System, Portland, Oregon; 2 Division of Infectious Diseases, Department of Medicine, Oregon Health and Sciences University, Portland; 3 Center for Clinical Management Research, VA Ann Arbor Health Care System, Ann Arbor, Michigan; 4 Center of Innovation to Improve Veteran Involvement in Care, VA Portland Health Care System, Portland, Oregon; 5 Health Management and Policy, School of Social and Behavioral Health Sciences, College of Public Health and Human Sciences, Oregon State University, Corvallis; 6 Health Data and Informatics Program, Center for Quantitative Life Sciences, Oregon State University, Corvallis; 7 Veterans Affairs Center for Medication Safety - Pharmacy Benefit Management (PBM) Services, Hines, Illinois; 8 Department of Anesthesiology, University of Michigan, Ann Arbor; 9 Seattle Epidemiologic Research and Information Center, Veterans Affairs Puget Sound Health Care System, Seattle, Washington; 10 Department of Internal Medicine, University of Michigan Medical School, Ann Arbor; 11 Center of Innovation to Accelerate Discovery and Practice Transformation, Durham VA Medical Center, Durham, North Carolina; 12 Department of Population Health Sciences, Duke University School of Medicine, Durham, North Carolina; 13 Duke-Margolis Center for Health Policy, Duke University, Durham, North Carolina; 14 Veterans Affairs Cooperative Studies Program Clinical Epidemiology Research Center (CSP-CERC), Veterans Affairs Connecticut Health Care System, West Haven; 15 Department of Biostatistics, Yale School of Public Health, New Haven, Connecticut; 16 Department of Medicine, Yale School of Medicine, New Haven, Connecticut; 17 Office of Research and Development, Veterans Health Administration, Washington, DC; 18 Divisions of Gastroenterology, Veterans Affairs Puget Sound Health Care System and University of Washington, Seattle, Washington; 19 Research and Development, Veterans Affairs Puget Sound Health Care System, Seattle, Washington

## Abstract

**Importance:**

Older adults and individuals with medical comorbidities are at increased risk for severe COVID-19. Several pharmacotherapies demonstrated to reduce the risk of COVID-19–related hospitalization and death have been authorized for use.

**Objective:**

To describe factors associated with receipt of outpatient COVID-19 pharmacotherapies in the Veterans Affairs (VA) health care system.

**Design, Settings, and Participants:**

This cohort study assessed outpatient veterans with risk factors for severe COVID-19 who tested positive for SARS-CoV-2 during January and February 2022. The setting was the VA health care system, the largest integrated health care system in the US.

**Exposures:**

Demographic characteristics, place of residence, underlying medical conditions, and COVID-19 vaccination.

**Main Outcomes and Measures:**

The odds of receipt of any COVID-19 pharmacotherapy, including sotrovimab, nirmatrelvir boosted with ritonavir, molnupiravir, or remdesivir were estimated using multivariable logistic regression.

**Results:**

Among 111 717 veterans included in this study (median [IQR] age, 60 [46-72] years; 96 482 [86.4%] male, 23 362 [20.9%] Black, 10 740 [9.6%] Hispanic, 75 973 [68.0%] White) who tested positive for SARS-CoV-2 during January to February 2022, 4233 (3.8%) received any COVID-19 pharmacotherapy, including 2870 of 92 396 (3.1%) in January and 1363 of 19 321 (7.1%) in February. Among a subset of 56 285 veterans with documented COVID-19–related symptoms in the 30 days preceding a positive SARS-CoV-2 test, 3079 (5.5%) received any COVID-19 pharmacotherapy. Untreated veterans had a median (IQR) age of 60 (46-71) years and a median (IQR) of 3 (2-5) underlying medical conditions. Veterans receiving any treatment were more likely to be older (aged 65 to 74 years vs 50 to 64 years: adjusted odds ratio [aOR], 1.66 [95% CI, 1.52-1.80]; aged at least 75 years vs 50 to 64 years: aOR, 1.67 [95% CI, 1.53-1.84]) and have a higher number of underlying conditions (at least 5 conditions vs 1 to 2 conditions: aOR, 2.17 [95% CI, 1.98-2.39]). Compared with White veterans, Black veterans (aOR, 0.65 [95% CI, 0.60-0.72]) were less likely to receive treatment; and compared with non-Hispanic veterans, Hispanic veterans (aOR, 0.88 [95% CI, 0.77-0.99]) were less likely to receive treatment. There were 16 546 courses of sotrovimab, nirmatrelvir, and molnupiravir allocated across the VA during this period.

**Conclusions and Relevance:**

In this cohort study of veterans who tested positive for SARS-CoV-2 during January and February when supply of outpatient COVID-19 pharmacotherapies was limited, prescription of these pharmacotherapies was underused, and many veterans with risk factors for severe COVID-19 did not receive treatment. Veterans from minority racial and ethnic groups were less likely to receive any pharmacotherapy.

## Key Points

**Question:**

How have antiviral agents and monoclonal antibodies for mild to moderate COVID-19 been used in the Veterans Affairs health care system?

**Findings:**

In this cohort study of 111 717 outpatient US veterans with clinical risk factors for severe COVID-19 who tested positive for SARS-CoV-2 during January and February 2022, 4233 (3.8%) received outpatient pharmacotherapy. Black veterans and Hispanic veterans were less likely to receive treatment, whereas older veterans with a higher number of underlying conditions were more likely to receive treatment.

**Meaning:**

These findings suggest that during a 2-month period when 4 anti–SARS-CoV-2 pharmacotherapies were authorized for use, few eligible veterans received treatment.

## Teaser

This cohort study examines factors associated with receipt of outpatient COVID-19 pharmacotherapies in the Veterans Affairs (VA) health care system among veterans with increased risk for severe COVID-19.

## Introduction

During January 2022 when the incidence of COVID-19 in the United States was at its highest, 82% of intensive care unit hospital beds were occupied, and nearly 900 000 COVID–19-related deaths had occurred since the pandemic began.^1,2^ Older adults and persons with underlying medical conditions such as chronic kidney disease, diabetes, and obesity are at increased risk for severe outcomes including hospitalization or death.^3^ Several neutralizing monoclonal antibodies and antivirals directed at SARS-CoV-2 have received US Food and Drug Administration (FDA) Emergency Use Authorization (EUA) for treatment of persons with mild to moderate COVID-19 who are at high risk for progression to severe disease.^4,5,6,7,8^ Most recently, these include EUA for nirmatrelvir boosted with ritonavir and molnupiravir in late December 2021 and remdesivir for outpatient use in January 2022. Although these therapies have been demonstrated in clinical trials to be effective in reducing the short-term risk of hospitalization or death among unvaccinated individuals,^9,10,11,12^ early limited drug supply, the requirement for prompt recognition of symptomatic disease and linkage to treatment, logistical barriers to administration, and the need for clinician and public awareness of therapeutic options have hampered widespread use.^13,14^ To date, use of outpatient SARS-CoV-2 pharmacotherapies in the US has not been well described.

The Veterans Affairs health care system (VA) is the largest integrated health care system in the US, providing care to more than 9 million veterans at 171 VA medical centers (VAMCs) and 1113 outpatient sites of care.^15^ Within the VA, COVID-19 pharmacotherapies allocated by the federal government are distributed across 156 VA pharmacies by the Pharmacy Benefits Management Services (PBM). This national distribution system serving a population with a majority of older adults with a high burden of underlying conditions who are frequently at increased risk for severe COVID-19^16^ provides a unique opportunity to evaluate how these therapies have been allocated to at-risk patients infected with SARS-CoV-2, including among minority racial and ethnic groups for whom reach of novel pharmacotherapies in the general US population is often unequal.^17^ Thus, we sought to describe rates and factors associated with prescription of outpatient COVID-19 pharmacotherapies during January and February 2022 when sotrovimab, a monoclonal antibody active against circulating Omicron SARS-CoV-2 variants at the time and antivirals nirmatrelvir, molnupiravir, and remdesivir were authorized for use.^8^

## Methods

### Study Setting and Data Sources

We used the VA’s COVID-19 Shared Data Resource (CSDR),^18^ supported by the VA Informatics and Computing Infrastructure (VINCI) which integrates multiple data sources to provide patient-level COVID-19–related information on VA enrollees. CSDR includes information on first laboratory-confirmed SARS-CoV-2 tests (either by nucleic acid amplification or antigen testing) within the VA system as well as tests performed outside the VA but documented in VA clinical records. Positive tests are identified by the VA National Surveillance Tool^19^ and provisioned to the CSDR to support national VA research and operational needs. We also used the VA Corporate Data Warehouse (CDW), a nationally linked database of the VA electronic health records system (EHR), which contains medical and administrative data as well as pharmacy records. These data were supplemented with detailed claims data (primarily receipt of COVID-19 monoclonal antibodies) from the VA Community Care program, which coordinates and reimburses local care provided outside the VA. We included linked data from the Centers for Medicare and Medicaid Services (CMS) to enhance capture of information on COVID-19 vaccination occurring in non–VA settings. We also integrated data from PBM surveillance on the allocation of sotrovimab, nirmatrelvir, and molnupiravir across VA pharmacies. Remdesivir was approved by the FDA in October 2020 for hospitalized patients with COVID-19 and therefore not part of the national PBM distribution of EUA pharmacotherapies.^20^ The study was approved by the VA Puget Sound and VA Portland institutional review boards and followed the Strengthening the Reporting of Observational Studies in Epidemiology (STROBE) reporting guideline.

### Study Population and Baseline Characteristics

We identified veterans aged 18 years or older with a first positive laboratory-confirmed SARS-CoV-2 test in CSDR between January 1 and February 28, 2022. We limited the study population to VA enrollees with an inpatient or outpatient encounter in the VA health care system in the 18 months preceding January 1, 2022, who were not hospitalized on or before the date of positive SARS-CoV-2 test (and hence considered to have mild to moderate COVID-19). We further limited test-positive patients to veterans with at least 1 risk factor for progression to severe COVID-19, including hospitalization or death, as defined by the FDA and the US Centers for Disease Control and Prevention (CDC) (eTable 1 in the Supplement).^3,4,5,21^

Using the date of the positive SARS-CoV-2 test as the index date, we ascertained baseline demographic characteristics including race and ethnicity (associated with COVID-19 care) as reported in the VA EHR and most recent ZIP code of residence documented in the year prior to the index date. The ZIP code was used to determine Veterans Integrated Services Network (VISN), rurality of residence based on the Rural-Urban Commuting Areas (RUCA) system, and distance to the nearest VAMC allocated COVID-19 pharmacotherapies.^22,23^ We also determined COVID-19 vaccination status (eMethods in the Supplement), current or former smoking, any alcohol or other substance dependence in the 2 years prior, underlying medical conditions documented within the last 2 years (eTable 1 in the Supplement), recent receipt of immunosuppressive medications or cancer therapies (eTable 2 in the Supplement), and National Institutes of Health (NIH) tier of prioritization for anti-SARS-CoV-2 therapies.^24^ Finally, we ascertained 15 prespecified COVID-19–related symptoms present on the index date or within the preceding 30 days, as available in CSDR.^18^ These symptoms were extracted from EHR *International Statistical Classification of Diseases and Related Health Problems, Tenth Revision (ICD-10)* codes, vital signs (temperature), and VINCI-generated natural language processing of admission diagnoses, COVID-19 symptom screening questionnaires, and relevant clinical notes.

### COVID-19 Pharmacotherapies

We determined receipt of 4 FDA authorized therapies in use between January 1 and February 28, 2022, which included sotrovimab, nirmatrelvir, molnupiravir, and remdesivir as captured by prescriptions within the VA as well as VA community care claims for reimbursed care provided outside of the VA. To distinguish outpatient from inpatient remdesivir use, we reviewed the date of first remdesivir dose relative to the date of hospital admission. Veterans who were not hospitalized after the index date, who received their first dose of remdesivir prior to any hospitalization, or who received their first dose while in the hospital where the length of stay lasted less than 1 day were classified as having received outpatient remdesivir.

### Statistical Analysis

Among veterans included in our study, we described sociodemographic and clinical characteristics, stratified by receipt of sotrovimab, nirmatrelvir, molnupiravir, remdesivir, or no antiviral nor monoclonal antibody (no treatment). Among treated veterans, we also described the relative proportion of each of the 4 pharmacotherapies prescribed by VISN. We conducted binomial logistic regression to estimate odds ratios for receipt of any pharmacotherapy vs no treatment as well as multinomial and binomial logistic regression to estimate odds ratios for receipt of sotrovimab, nirmatrelvir, molnupiravir, or remdesivir vs no treatment. Base models for each factor of interest were adjusted for age, sex, and race and ethnicity. Additional covariates identified a priori as potential confounders based on clinical and organizational knowledge (VISN, rural residence, underlying conditions, tobacco, alcohol, substance use) were included for each comparison if they changed the adjusted odds ratio (aOR) by at least 5% when added individually to each base model. Final models were limited to veterans with complete data for all included covariates. Odds ratios were compared using 95% CIs. Analyses were conducted using SAS Enterprise Guide 8.2 (SAS Institute) from April to June 2022.

## Results

### Descriptive Results

**Figure 1.**
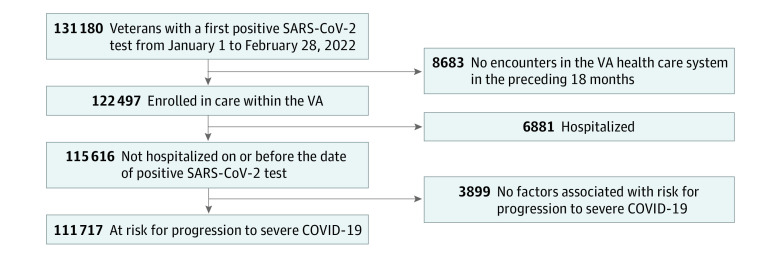
Study Flowchart Veterans with a first positive SARS-CoV-2 test from January 1-February 28, 2022, were included in the study. Eligible veterans had an inpatient or outpatient encounter in the VA health care system in the 18 months preceding January 1, 2022, were not hospitalized on or before the date of positive SARS-CoV-2 test, and had at least 1 risk factor for progression to severe COVID-19.

During January and February 2022, 111 717 VA enrollees (median [IQR] age, 60 [46-72] years; 96 482 [86.4%] male, 23 362 [20.9%] Black, 10 740 [9.6%] Hispanic, 75 973 [68.0%] White) with a first positive SARS-CoV-2 test were eligible to receive therapy for mild to moderate COVID-19 (Figure 1), of whom 4233 (3.8%) received any pharmacotherapy within the VA or through VA Community Care (Table 1). This included 994 (0.9%) persons who received sotrovimab, 1710 (1.5%) who received nirmatrelvir, 921 (0.8%) who received molnupiravir, 608 (0.5%) who received remdesivir and 107 484 (96.2%) who were not treated with anti–SARS-CoV-2 pharmacotherapies within the VA or through VA Community Care; 2870 of 92 396 veterans (3.1%) diagnosed in January and 1363 of 19 321 veterans (7.1%) diagnosed in February received any of the pharmacotherapies of interest. Among the subset of 53 206 veterans with any COVID-19–related symptoms, 3079 (5.5%) received any pharmacotherapy, whereas 1384 of 37 274 veterans (3.4%) who had not completed primary or booster COVID-19 vaccination received any pharmacotherapy. There were an estimated 16 546 total courses of sotrovimab (n = 3770), nirmatrelvir (n = 5220), and molnupiravir (n = 7556) distributed across the VA; 3625 courses (21.9%) were prescribed to eligible veterans, including 26.4% of the available sotrovimab supply, 32.8% of nirmatrelvir, and 12.2% of molnupiravir.

**Table 1.** Characteristics of Veterans Testing Positive for SARS-CoV-2 by Receipt of Outpatient COVID-19 Pharmacotherapy, January and February 2022

Characteristic	Veterans, No. (%)						
	All veterans (N = 111 717)^a^	No treatment (n = 107 484)	Any treatment (n = 4233)	Sotrovimab (n = 994)	Nirmatrelvir and ritonavir (n = 1710)	Molnupiravir (n = 921)	Remdesivir (n = 608)
Age, median (IQR), y	60 (46-72)	60 (46-71)	68 (58-74)	71 (63-75)	66 (54-74)	68 (57-75)	68 (59-75)
Age group, y							
18-49	33 206 (29.7)	32 646 (30.4)	560 (13.2)	86 (8.7)	293 (17.1)	113 (12.3)	68 (11.2)
50-64	33 976 (30.4)	32 849 (30.6)	1127 (26.6)	193 (19.4)	501 (29.3)	254 (27.6)	179 (29.4)
65-74	26 971 (24.1)	25 464 (23.7)	1507 (35.6)	416 (41.9)	566 (33.1)	323 (35.1)	202 (33.2)
≥75	17 564 (15.7)	16 525 (15.4)	1039 (24.5)	299 (30.1)	350 (20.5)	231 (25.1)	159 (26.2)
Male sex	96 482 (86.4)	92 648 (86.2)	3834 (90.6)	911 (91.6)	1527 (89.3)	836 (90.8)	560 (92.1)
Race^b^							
Black	23 362 (20.9)	22 740 (21.2)	622 (14.7)	127 (12.8)	252 (14.7)	137 (14.9)	106 (17.4)
White	75 973 (68)	72 726 (67.7)	3247 (76.7)	769 (77.4)	1318 (77.1)	721 (78.3)	439 (72.2)
Other	3896 (3.5)	3783 (3.5)	113 (2.7)	34 (3.4)	43 (2.5)	20 (2.2)	16 (2.6)
Ethnicity^c^							
Not Hispanic	95 607 (85.6)	91 870 (85.5)	3737 (88.3)	888 (89.3)	1492 (87.3)	828 (89.9)	529 (87)
Hispanic	10 740 (9.6)	10 414 (9.7)	326 (7.7)	70 (7)	156 (9.1)	54 (5.9)	46 (7.6)
Rurality^d^							
Urban	94 217 (84.3)	90 832 (84.5)	3385 (80)	775 (78)	1396 (81.6)	717 (77.9)	497 (81.7)
Rural	17 500 (15.7)	16 652 (15.5)	848 (20)	219 (22)	314 (18.4)	204 (22.1)	111 (18.3)
Region^e^							
West	27 931 (25)	27 012 (25.1)	919 (21.7)	209 (21)	366 (21.4)	224 (24.3)	120 (19.7)
Midwest	20 451 (18.3)	19 522 (18.2)	929 (21.9)	256 (25.8)	422 (24.7)	208 (22.6)	43 (7.1)
Northeast	15 432 (13.8)	14 642 (13.6)	790 (18.7)	208 (20.9)	339 (19.8)	97 (10.5)	146 (24)
South	47 903 (42.9)	46 308 (43.1)	1595 (37.7)	321 (32.3)	583 (34.1)	392 (42.6)	299 (49.2)
Distance from place of residence to nearest VAMC facility allocated the respective product, miles, median (IQR)^f^	18 (8-39)	18 (8-39)	17 (8-34)	20 (8-41)	16 (7-31)	16 (7-32)	N/A
Month of positive test							
January	92 396 (82.7)	89 526 (83.3)	2870 (67.8)	651 (65.5)	1064 (62.2)	647 (70.2)	508 (83.6)
February	19 321 (17.3)	17 958 (16.7)	1363 (32.2)	343 (34.5)	646 (37.8)	274 (29.8)	100 (16.4)
≥1 symptom in the 30 d prior to positive SARS-CoV-2 test	56 285 (50.4)	53 206 (49.5)	3079 (72.7)	707 (71.1)	1228 (71.8)	696 (75.6)	448 (73.7)
Vaccination status^g^							
No vaccination	34 258 (30.7)	33 108 (30.8)	1150 (27.2)	276 (27.8)	500 (29.2)	212 (23)	162 (26.6)
Partial vaccination	4369 (3.9)	4135 (3.8)	234 (5.5)	69 (6.9)	84 (4.9)	37 (4)	44 (7.2)
Primary vaccination	46 079 (41.2)	44 482 (41.4)	1597 (37.7)	402 (40.4)	616 (36)	361 (39.2)	218 (35.9)
Booster vaccination	26 980 (24.2)	25 728 (23.9)	1252 (29.6)	247 (24.8)	510 (29.8)	311 (33.8)	184 (30.3)
Other	31 (0)	31 (0)	0 (0)	0 (0)	0 (0)	0 (0)	0 (0)
NIH Tier^h^							
1	14 987 (13.4)	13 913 (12.9)	1074 (25.4)	385 (38.7)	360 (21.1)	161 (17.5)	168 (27.6)
2	25 559 (22.9)	24 985 (23.2)	574 (13.6)	83 (8.4)	294 (17.2)	118 (12.8)	79 (13)
3	31 995 (28.6)	30 313 (28.2)	1682 (39.7)	398 (40)	640 (37.4)	411 (44.6)	233 (38.3)
4	39 148 (35)	38 245 (35.6)	903 (21.3)	128 (12.9)	416 (24.3)	231 (25.1)	128 (21.1)
Smoking^i^							
Never	43 676 (39.1)	42 085 (39.2)	1591 (37.6)	355 (35.7)	673 (39.4)	329 (35.7)	234 (38.5)
Former	44 506 (39.8)	42 626 (39.7)	1880 (44.4)	485 (48.8)	720 (42.1)	411 (44.6)	264 (43.4)
Current	19 057 (17.1)	18 454 (17.2)	603 (14.2)	112 (11.3)	255 (14.9)	156 (16.9)	80 (13.2)
Alcohol dependence							
Never	86 379 (77.3)	82 856 (77.1)	3523 (83.2)	845 (85)	1414 (82.7)	762 (82.7)	502 (82.6)
Ever	25 338 (22.7)	24 628 (22.9)	710 (16.8)	149 (15)	296 (17.3)	159 (17.3)	106 (17.4)
Substance dependence							
Never	105 845 (94.7)	101 778 (94.7)	4067 (96.1)	965 (97.1)	1651 (96.5)	881 (95.7)	570 (93.8)
Ever	5872 (5.3)	5706 (5.3)	166 (3.9)	29 (2.9)	59 (3.5)	40 (4.3)	38 (6.3)
No. of underlying conditions, median (IQR)	3 (2-5)	3 (2-5)	4 (3-14)	5 (3-15)	4 (2-7)	4 (3-14)	5 (3-15)
Charlson Comorbidity Index, mean	1.5	1.5	2.4	3.2	1.9	2.4	2.7
Underlying condition							
Obese (body mass index ≥30 kg/m^2^)	56 708 (50.8)	54 535 (50.7)	2173 (51.3)	505 (50.8)	886 (51.8)	497 (54)	285 (46.9)
Chronic kidney disease	13 594 (12.2)	12 749 (11.9)	845 (20)	319 (32.1)	216 (12.6)	192 (20.8)	118 (19.4)
Diabetes	33 543 (30)	31 718 (29.5)	1825 (43.1)	508 (51.1)	664 (38.8)	386 (41.9)	267 (43.9)
Immunosuppressive medications or cancer therapies^j^	4760 (4.3)	4242 (3.9)	518 (12.2)	211 (21.2)	150 (8.8)	63 (6.8)	94 (15.5)
Cancer	20 150 (18)	18 967 (17.6)	1183 (27.9)	350 (35.2)	448 (26.2)	229 (24.9)	156 (25.7)
Cardiovascular disease	67 149 (60.1)	63 949 (59.5)	3200 (75.6)	829 (83.4)	1193 (69.8)	712 (77.3)	466 (76.6)
Chronic lung disease	23 096 (20.7)	21 817 (20.3)	1279 (30.2)	349 (35.1)	447 (26.1)	288 (31.3)	195 (32.1)
Dementia	3889 (3.5)	3656 (3.4)	233 (5.5)	54 (5.4)	69 (4)	44 (4.8)	66 (10.9)
Cerebrovascular disease	5579 (5)	5236 (4.9)	343 (8.1)	91 (9.2)	93 (5.4)	88 (9.6)	71 (11.7)
Chronic liver disease	9642 (8.6)	9191 (8.6)	451 (10.7)	125 (12.6)	160 (9.4)	88 (9.6)	78 (12.8)
Mental health conditions^k^	46 349 (41.5)	44 729 (41.6)	1620 (38.3)	345 (34.7)	622 (36.4)	386 (41.9)	267 (43.9)

Abbreviation: NIH, National Institutes of Health.

^a^ Among veterans enrolled in care, not hospitalized at the time of positive test, and with at least 1 risk factor for severe COVID-19.

^b^ Race unknown: 8486 (8%) in the all-veterans group, 8235 (8%) in the no-treatment group, 251 (6%) in any treatment group, 64 (6%) who received sotrovimab, 97 (6%) who received nirmaltrelvir, 43 (5%) who received molnupiravir, and 47 (8%) who received remdesivir. Other race includes Asian, American Indian or Alaska Native, Native Hawaiian or Pacific Islander.

^c^ Ethnicity unknown: 5370 (5%) in the all-patient group, 5200 (5%) in the no-treatment group, 170 (4%) in any treatment group, 36 (4%) who received sotrovimab, 62 (4%) who received nirmaltrelvir, 39 (4%) who received molnupiravir, and 33 (5%) who received remdesivir.

^d^ Based on rural-urban commuting area (RUCA) codes.

^e^ Regions are based on Veterans Integrated Service Networks (VISNs). West includes VISNs 19-22; Midwest: 10, 12, 15, 23; Northeast: 1, 2, 4, 5; South: 6-9, 16-17.

^f^ For the no treatment group, distance is calculated to the nearest facility prescribing any treatment.

^g^ See eAppendix in the Supplement.

^h^ As defined by the NIH. See eTable 3 in the Supplement.

^i^ Smoking unknown: 4478 (4%) in the all-patient group, 4319 (4%) in the no-treatment group, 159 (4%) in any treatment group, 42 (4%) who received sotrovimab, 62 (4%) who received nirmaltrelvir, 25 (3%) who received molnupiravir, and 30 (5%) who received remdesivir.

^j^ See eTable 2 in the Supplement.

^k^ Includes major depressive disorder, bipolar disorder, schizophrenia.

Among untreated veterans, median (IQR) age was 60 (46-71) years, 22 740 (21.2%) were Black, 72 726 (67.7%) were White, 70 210 (65.3%) had completed primary or booster COVID-19 vaccination, and median (IQR) number of underlying conditions was 3 (2-5) (Table 1). Among persons treated with sotrovimab, median (IQR) age was 71 (63-75) years, 127 (12.8%) were Black, 767 (77.4%) were White, 649 (65.2%) had received full or boosted COVID-19 vaccination, and median (IQR) number of underlying conditions was 5 (3-15). Among persons treated with nirmatrelvir, median (IQR) age was 66 (54-74) years, 252 (14.7%) were Black, 1318 (77.1%) were White, 1116 (65.8%) had received full or boosted COVID-19 vaccination, and median (IQR) number of underlying conditions was 4 (2-7). Among persons treated with molnupiravir, median (IQR) age was 68 (57-75) years, 137 (14.9%) were Black, 721 (78.3%) were White, 672 (73.0%) had received full or boosted COVID-19 vaccination, and median (IQR) number of underlying conditions was 4 (3-14). Among persons treated with remdesivir, median (IQR) age was 68 (59-75) years, 106 (17.4%) were Black, 439 (72.2%) were White, 402 (66.2%) had received full or boosted COVID-19 vaccination, and median (IQR) number of underlying conditions was 5 (3-15). Veterans who received any treatment lived a median (IQR) 17 (8-34) miles from the nearest dispensing VAMC, while untreated veterans lived a median (IQR) 18 (8-39) miles from the nearest dispensing facility.

### Regression Results

Veterans receiving any of the 4 treatments were more likely to be older (aged 65 to 74 years vs 50 to 64 years: aOR, 1.66 [95% CI, 1.52-1.80]; aged at least 75 years vs 50 to 64 years: aOR, 1.67 [95% CI, 1.53-1.84]), live in rural areas (aOR, 1.18 [95% CI, 1.09-1.28]), and have a higher number of underlying conditions (at least 5 conditions vs 1 to 2 conditions: aOR, 2.17 [95% CI, 1.98-2.39]) (Table 2). Receipt of immunosuppressive medications or cancer treatments was associated with treatment (aOR, 3.03 [95% CI, 2.74-3.36]). Compared with White veterans, Black veterans (aOR, 0.65 [95% CI, 0.60-0.72]) were less likely to receive treatment; and compared with non-Hispanic veterans, Hispanic veterans (aOR, 0.88 [95% CI, 0.77-0.99]) were less likely to receive treatment. Veterans with alcohol dependence were less likely to receive treatment than those with no alcohol dependence (aOR, 0.79 [95% CI, 0.72-0.86]). Veterans partially vaccinated against COVID-19 were more likely to receive any treatment compared with unvaccinated veterans (aOR, 1.52 [95% CI, 1.30-1.77]), whereas there were no significant differences among fully vaccinated (aOR, 0.96 [95% CI, 0.89-1.04]) or boosted persons (aOR, 1.06 [95% CI, 0.97-1.16]). Factors associated with receipt of any treatment were similar when evaluated by individual pharmacotherapies (eTable 4 in the Supplement) or when restricting to symptomatic individuals (eTable 5 in the Supplement).

**Table 2.** Factors Associated With Receipt of Any COVID-19 Pharmacotherapy Among Veterans, January and February 2022

Characteristic	Any pharmacotherapy^a^		
	No./total No. (%)^b^	Unadjusted odds ratio (95% CI)	Adjusted odds ratio^c^ (95% CI)
Age, y			
18-49	507/28 890 (1.8)	0.50 (0.45-0.55)	0.51 (0.46-0.57)
50-64 (reference)	1030/30 749 (3.4)	NA	NA
65-74 (reference)	1401/24 815 (5.7)	1.72 (1.59-1.87)	1.66 (1.52-1.80)
≥75	952/16 133 (5.9)	1.83 (1.68-2.00)	1.67 (1.53-1.84)
Female sex	359/13 526 (2.7)	0.65 (0.59-0.72)	0.97 (0.86-1.09)
Race			
Black	606/22 756 (2.7)	0.61 (0.56-0.67)	0.65 (0.60-0.72)
Hispanic ethnicity	272/8609 (3.2)	0.77 (0.69-0.86)	0.88 (0.77-0.99)
White (reference)	3179/74 093 (4.3)	NA	NA
Other	105/3738 (2.8)	0.67 (0.55-0.81)	0.76 (0.62-0.93)
Rural residence	791/16 137 (4.9)	1.37 (1.27-1.48)	1.18 (1.09-1.28)
Region			
West (reference)	822/23 959 (3.4)	NA	NA
Midwest	872/18 831 (4.6)	1.40 (1.27-1.54)	1.32 (1.20-1.46)
Northeast	732/14 147 (5.2)	1.59 (1.44-1.75)	1.51 (1.36-1.67)
South	1464/43 650 (3.4)	1.01 (0.93-1.10)	1.03 (0.94-1.12)
COVID-19 vaccination			
None (reference)	1045/30 671 (3.4)	NA	NA
Partial	209/3917 (5.3)	1.63 (1.41-1.88)	1.52 (1.30-1.77)
Primary	1476/41 418 (3.6)	1.03 (0.96-1.12)	0.96 (0.89-1.04)
Booster	1160/24 557 (4.7)	1.40 (1.29-1.52)	1.06 (0.97-1.16)
Smoking			
Never (reference)	1462/39 061 (3.7)	NA	NA
Former	1728/40 274 (4.3)	1.17 (1.09-1.25)	0.99 (0.92-1.07)
Current	562/17 286 (3.3)	0.86 (0.79-0.95)	0.90 (0.81-0.99)
Alcohol dependence	646/22 658 (2.9)	0.68 (0.62-0.74)	0.79 (0.72-0.86)
Substance dependence	154/5400 (2.9)	0.73 (0.62-0.85)	0.90 (0.76-1.06)
No. of underlying conditions			
1-2 (reference)	790/37 502 (2.1)	NA	NA
3-4	1222/31 289 (3.9)	1.89 (1.73-2.06)	1.63 (1.48-1.79)
≥5	1845/29 852 (6.2)	3.10 (2.86-3.36)	2.17 (1.98-2.39)
Cancer	1102/18 626 (5.9)	1.81 (1.69-1.94)	1.38 (1.29-1.49)
Cardiovascular disease	1790/30 052 (6.0)	2.08 (1.96-2.21)	1.52 (1.41-1.63)
Chronic kidney disease	783/12 539 (6.2)	1.85 (1.71-2.00)	1.40 (1.29-1.53)
Chronic lung disease	1558/28 957 (5.4)	1.71 (1.60-1.82)	1.39 (1.31-1.49)
Diabetes	1688/30 708 (5.5)	1.81 (1.70-1.93)	1.43 (1.33-1.53)
Immunosuppressive or cancer medications	471/4361 (10.8)	3.39 (3.08-3.74)	3.03 (2.74-3.36)
Mental health conditions	1497/41 717 (3.6)	0.87 (0.82-0.93)	1.13 (1.05-1.21)
Obese (body mass index ≥30)	2002/51 129 (3.9)	1.02 (0.95-1.08)	1.19 (1.11-1.27)

^a^ Includes sotrovimab, nirmatrelvir, molnupiravir, and remdesivir.

^b^ A total of 4233 veterans who received any COVID-19 pharmacotherapy out of 111 717 veterans testing positive for SARS-CoV-2 were included. Models were limited to veterans with complete data for all included covariates.

^c^ All models adjusted for age, sex, race, and ethnicity. Additional covariates did not change the adjusted odds ratio by at least 5% and were therefore not included in the final models.

**Figure 2.**
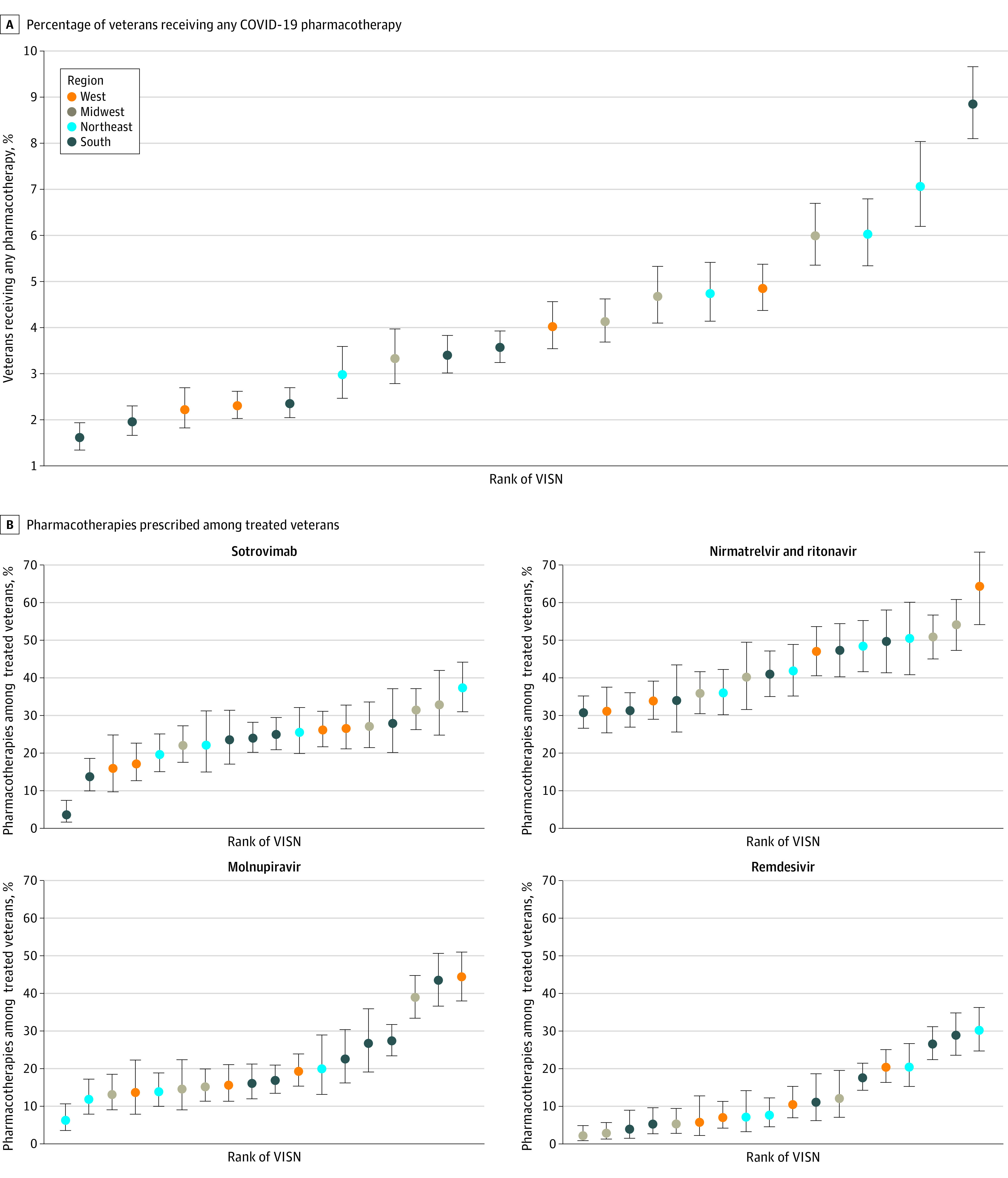
Distribution of COVID-19 Pharmacotherapies by Veterans Integrated Services Networks A, Percentage of veterans receiving any COVID-19 pharmacotherapy. B, Pharmacotherapies prescribed among treated veterans. Regions are based on VISNs. West includes VISNs 19 to 22; Midwest: 10, 12, 15, 23; Northeast: 1, 2, 4, 5; South: 6 to 9, 16, 17. Error bars indicate 95% CIs for proportions; VISN, Veterans Integrated Services Network.

Among veterans treated with anti–SARS-CoV-2 pharmacotherapies, the relative proportion of treatments varied across VISNs, from 3.5% to 37.3% for sotrovimab, 30.7% to 64.4% for nirmatrelvir, 6.5% to 44.7% for molnupiravir, and 2.4% to 30.5% for remdesivir (Figure 2).

## Discussion

In a nationwide study of 111 717 outpatient US veterans seen in the VA health care system who tested positive for SARS-CoV-2 in January and February 2022, only 3.8% received any outpatient pharmacotherapy, including 5.5% of those with COVID-19–related symptoms and 3.4% of those who were unvaccinated or only partially vaccinated. Untreated veterans during this early period included many persons at high risk for progression to severe COVID-19. Persons of Black race and Hispanic ethnicity were less likely to receive treatment whereas older veterans with a higher number of underlying conditions were more likely to receive treatment. There were notable geographic differences in the distribution of selected pharmacotherapies.

Prescription of outpatient SARS-CoV-2 pharmacotherapies following FDA EUA of oral antivirals in December 2021^8^ has not been well described. As reported here, even among at-risk patients engaged in care in a system with national distribution across 156 sites, use remained low. Because oral antivirals were authorized just before the start of the study period, it is expected for initial uptake to be low and increase with time as clinical familiarity and infrastructures develop; in fact, the proportion of veterans testing positive for SARS-CoV-2 who were treated more than doubled from 3.1% in January to 7.1% in February. Nonetheless, there was still a relative surplus in drug supply during this period. Low use is not unique to the VA^13,25^ and has been observed in the US and other countries^26,27,28^ reflecting widespread, complex barriers to optimal use.

Across VISNs, there were notable differences in the percentage of veterans receiving any COVID-19 pharmacotherapy as well the relative use of different pharmacotherapies, likely reflecting differences in local infrastructure and clinician education. Furthermore, in the absence of head-to-head clinical trials or observational study evidence for the effectiveness of authorized therapies during this early period, clinical practice varied. Local and regional differences in patient awareness of therapies, particularly during the period of this study before the launch of the nationwide COVID-19 Test to Treat Initiative,^29^ also likely contributed to variability in prescribing across VISNs.

Differences in treatment by rurality were also observed, with veterans in rural areas somewhat more likely to receive treatment. Although US Veterans are a highly rural population,^30^ many VA facilities allocated anti–SARS-CoV-2 pharmacotherapies are also located in rural areas. Rural veterans in our study, in addition to being older, male, and non-Hispanic White, also had more underlying conditions than their urban counterparts (data not shown). Veterans testing positive for SARS-CoV-2 lived within a relatively close distance of a dispensing facility, and we did not observe a meaningful difference between treated and untreated persons, suggesting that relative to other factors, physical distance may not have been a substantial barrier to treatment. However, veterans living farther from VA facilities are more likely to engage in VA care, which may have contributed to better ascertainment of prescriptions in this group and a higher observed likelihood of treatment among rural veterans.^31^

Consistent with findings in other studies, Black veterans were less likely to receive outpatient treatments for COVID-19; Hispanic veterans in our study were also slightly less likely to receive treatment, whereas other studies have been mixed.^13,25^ While demographic differences in access to care, including lower use of COVID-19 monoclonal antibody treatments among people from minority racial and ethnic groups have been well-described in non–veteran populations,^25^ disparities in COVID-19–related care within the VA system have been less pronounced.^16,32^ Possible reasons for observed racial and ethnic differences in the treatment of mild to moderate COVID-19 include lower awareness of COVID-19 therapy efficacy and eligibility, differences in care-seeking and access to primary care, transportation challenges, potential clinician biases, and lower trust in the health care system impacting acceptance of recommended investigational therapies.^14,25,33,34,35^

The association between increasing age and higher number of underlying conditions with receipt of pharmacotherapy is consistent with NIH patient prioritization recommendations, which include age, immune status, and clinical risk factors as key determinants for assessing risk for progression to severe COVID-19.^24^ NIH guidance also prioritizes unvaccinated individuals, however, we did not observe a consistent pattern between likelihood of treatment and COVID-19 vaccination; despite the early emphasis on treating unvaccinated individuals, we did not observe any differences in the likelihood of treatment in this group compared with fully vaccinated or boosted veterans. On the other hand, partially vaccinated veterans were more likely to be treated. These findings may reflect differences in patient behaviors, with veterans willing to seek vaccination also more likely to accept recommended investigational therapies. Differences in clinician practice may also affect observed results; because the demonstrated benefit of COVID-19 pharmacotherapies in clinical trials was among unvaccinated participants, clinicians may have differing perspectives on the potential of these therapies to reduce the risk of severe COVID-19 among vaccinated persons. With nearly two-thirds of veterans in our study fully vaccinated or boosted, evidence from observational studies for the effectiveness of current used therapies will be essential to inform best practices.

### Limitations

This study has several important limitations. Eligibility for treatment of mild to moderate COVID-19 under FDA EUA requires symptomatic disease, and we were not able to fully ascertain COVID-19–related symptoms. National surveillance is conducted by VA PBM to ensure eligibility among veterans receiving treatment in the VA; however, determination of symptom eligibility among untreated veterans is limited to use of natural language processing supplementation of structured data. Nonetheless, even with the study population restricted to symptomatic individuals, the percentage of veterans (5.5%) receiving treatment was still small. Although we augmented data from the VA EHR with multiple sources of information, including receipt of COVID-19 monoclonal antibodies through the VA Community Care program, it is still possible that veterans were treated for COVID-19 outside of the VA. However, this study was conducted during a time when treatments were still very limited. We also restricted the study population to veterans engaged in VA care to increase the likelihood that treatment would occur within the VA system. Although CSDR captures laboratory-based SARS-CoV-2 tests performed in the VA as well as non-VA testing documented in clinical notes, we did not identify all veterans testing positive for SARS-CoV-2, particularly those who did not report non-VA or home-based testing to their clinician. Thus, we may have underestimated the true number of untreated veterans. Furthermore, our estimates of prescribed pharmacotherapies do not account for veterans who were offered but declined treatment. Additionally, veterans are older and have more underlying conditions than persons in the general US population, and care delivery in the VA is very different from non–VA systems; therefore, usage findings may not be generalizable to other groups.

## Conclusions

In this nationwide study of US veterans using the VA health care system who tested positive for SARS-CoV-2 during January and February 2022, most eligible persons were not prescribed treatment for mild to moderate COVID-19. Demographic, clinical, and facility-level factors were associated with the likelihood of being prescribed treatment. These findings suggest the need for improved infrastructure and education to support treatment of persons at risk for progression to severe COVID-19. Studies of the effectiveness and comparative effectiveness of outpatient COVID-19 pharmacotherapies will also be critical to inform clinical care.

## Notes

Supplement. **eAppendix.** Supplemental Methods **eTable 1.** Risk Factors for Severe COVID-19 **eTable 2.** Immunosuppressive and Cancer Medications **eTable 3.** National Institutes of Health (NIH) Tiers of Prioritization for Anti-SARS-CoV-2 Therapies **eTable 4.** Factors Associated With Receipt of Different COVID-19 Pharmacotherapies Among Veterans, January-February 2022 **eTable 5.** Factors Associated With Receipt of Any COVID-19 Pharmacotherapy Among Symptomatic Veterans, January-February 2022
eReferences
