# Supplementary material for: Early Adoption of Anti–SARS-CoV-2 Pharmacotherapies Among US Veterans With Mild to Moderate COVID-19, January and February 2022
Source: JAMA Netw Open. 2022 Nov 11;5(11):e2241434. doi: 10.1001/jamanetworkopen.2022.41434 (PMC9652752; doi:10.1001/jamanetworkopen.2022.41434)
Supplement: Supplement. — eAppendix. Supplemental Methods eTable 1. Risk Factors for Severe COVID-19 eTable 2. Immunosuppressive and Cancer Medications eTable 3. National Institutes of Health (NIH) Tiers of Prioritization for Anti-SARS-CoV-2 Therapies eTable 4. Factors Associated With Receipt of Different COVID-19 Pharmacotherapies Among Veterans, January-February 2022 eTable 5. Factors Associated With Receipt of Any COVID-19 Pharmacotherapy Among Symptomatic Veterans, January-February 2022 eReferences [file jamanetwopen-e2241434-s001.pdf]

Supplemental Online Content

Bajema KL, Wang XQ, Hynes DM, et al. Early adoption of anti-SARS-CoV-2 pharmacotherapies among US veterans with mild to moderate COVID-19, January to February 2022. *JAMA Netw Open.* 2022;5(11):e2241434. doi:10.1001/jamanetworkopen.2022.41434

eAppendix. Supplemental Methods

eTable 1. Risk Factors for Severe COVID-19

eTable 2. Immunosuppressive and Cancer Medications

eTable 3. National Institutes of Health (NIH) Tiers of Prioritization for Anti-SARS-CoV-2 Therapies

eTable 4. Factors Associated With Receipt of Different COVID-19 Pharmacotherapies Among Veterans, January-February 2022

eTable 5. Factors Associated With Receipt of Any COVID-19 Pharmacotherapy Among Symptomatic Veterans, January-February 2022

eReferences

This supplemental material has been provided by the authors to give readers additional information about their work.

eAppendix. Supplemental Methods

COVID-19 Vaccination Status

Veterans were considered unvaccinated if they did not receive any COVID-19 vaccine or received a vaccine dose other than Janssen less than 14 days prior to the first positive SARS-CoV-2 test (index date). Partial vaccination was defined as having received a single mRNA dose (Pfizer-BioNTech or Moderna) alone or in combination with another vaccine other than Janssen ≤14 days prior to the index date or a Janssen (Johnson & Johnson) dose <14 days before the index date. For Veterans not considered to be moderately or severely immunocompromised (i.e., had not recently received immunosuppressive or cancer medications as described in Supplementary Table 2), full vaccination was defined as receipt of both doses of an mRNA vaccine (Pfizer-BioNTech or Moderna) or a single Janssen dose ≥14 days before the index date. Non-immunocompromised Veterans were considered boosted if they either received 1 dose of Janssen followed by another dose of Janssen or mRNA vaccine ≥14 days before the index date or if they received ≥3 vaccine doses (which included at least 1 mRNA dose) ≥7 days before the index date. Moderately or severely immunocompromised Veterans who either received 3 mRNA vaccine doses ≥7 days before the index date or 1 dose of Janssen followed by another dose of Janssen or mRNA vaccine ≥14 days before the index date were considered fully vaccinated.[1] Immunocompromised Veterans who received ≥4 mRNA doses ≥7 days before the index date were considered boosted.

NIH Tiers

To categorize vaccination as a binary variable for the National Institutes of Health (NIH) tiers of prioritization for anti-SARS-CoV-2 therapies, we considered unvaccinated and partially vaccinated Veterans as ‘unvaccinated’ for tiers 1 and 2 and fully vaccinated and boosted as ‘vaccinated.’

© 2022 Bajema KL et al. *JAMA Network Open*

eTable 1. Risk factors for severe COVID-19[a]

**Age ≥ 65 years**
**Underlying Medical Conditions**
Cancer
Cardiovascular disease including cardiomyopathy, chronic rheumatic heart disease, congestive heart failure, coronary artery disease, hypertension, myocardial infarction, peripheral artery disease, pulmonary heart disease
Chronic kidney disease including dialysis
Chronic liver disease including chronic hepatitis and cirrhosis
Chronic lung disease including asthma, chronic obstructive pulmonary disease, emphysema, pulmonary fibrosis
Chronic neurologic conditions including epilepsy, Multiple Sclerosis, and Parkinson's Disease
Dementia
Diabetes
HIV
Immunosuppressive medications or cancer therapies (see Supplementary Table 2)
Mental health conditions including bipolar disorder, major depressive disorder, and schizophrenia
Overweight (body mass index 25 to <30 kb/m^2) or obese (body mass index ≥30 kg/m^2) or
Pregnancy
Sickle cell disease
Stroke or cerebrovascular disease
Thalassemia
**Current or former tobacco use**
**Substance use**
Alcohol dependence
Non-alcohol substance dependence

[a]Age at the time of positive SARS-CoV-2 test or underlying conditions, tobacco, or substance use documented in the two years prior to positive SARS-CoV-2 test.

© 2022 Bajema KL et al. *JAMA Network Open*

eTable 2. Immunosuppressive and cancer medications[a]

Drug class	Drugs
Alkylating agents	bendamustine, busulfan, chlorambucil, cisplatin, cyclophosphamide, ifosfamide, lurbinectedin, melphalan, nitrogen mustard procarbazine, temozolomide
Anti-CD19/CD3	blinatumomab
Anti-HER2	trastuzumab
Antimetabolites	azathioprine, capecitabine, cladribine, cytarabine, fludarabine, floxuridine, fluorouracil, gemcitabine, mercaptopurine, methotrexate, pemtrexed, pentostatin, pralatrexate, thioguanine
Antitumor antibiotics	bleomycin, dacarbazine, dactinomycin, daunorubicin, doxorubicin, epirubidin, idarubicin, mitomycin, mitoxantrone
B-cell depletion and inhibition	belimumab, ibriumomab, tiuxetan, obinutuzumab, ocrelizumab,[b] ofatumumab,[b] rituximab[b]
BCL2 inhibitors	venetoxlax
Interferon	interferon alpha-2a, interferon alpha-2b, interferon beta-1a, interferon beta-1b, interferon gamma, interferon gamma-1b
BCR-ABL tyrosine kinase inhibitors	bosutinib, dasatinib, imatinib, nilotinib, ponatinib
Bruton tyrosine kinase (BTK) inhibitors	acalabrutinib, ibrutinib, zanubrutinib
Calcineurin inhibitors	cyclosporine, pimecrolimus, tacrolimus, voclosporin
Complement inhibitor	eculizumab
EGFR inhibitors	afatinib, cetuximab, dacomitinib, erlotinib, gefitinib, lapatinib, mobocertinib, necitumumab, osimertinib, panitumumab
Glucocorticoids (systemic)	cortisone acetate, betamethasone, budesonide, dexamethasone, hydrocortisone, methylprednisolone, prednisolone, prednisone
Histone deacetylase inhibitors	belinostat, vorinostat
IL-1 inhibitors	anakinra, canakinumab, rilonacept
IL-12/23 inhibitors	guselkumab, ustekinumab
IL-17 inhibitors	brodalumab, ixekizumab, secukinumab
IL-4/13 inhibitors	dupilumab
IL-5 inhibitors	benralizumab, mepolizumab, reslizumab
IL-6 inhibitors	sarilumab, siltuximab, tocilizumab
Immune checkpoint inhibitors (anti-CTLA-4)	ipilimumab
Immune checkpoint inhibitors (anti-PD-1)	cemiplimab, dostarlimab, nivolumab, pembrolizumab
Immune checkpoint inhibitors (anti-PD-L1)	atezolizumab, avelumab, durvalumab
Immune globulin	anti-thymocyte globulin[b]
Immunosuppressant agent	mycophenolate mofetil (MMF), mycophenolate sodium
Janus kinase (JAK) inhibitors	baricitinib, ruxolitinib, tofacitinib, upadacitinib
Miscellaneous antineoplastics	arsenic trioxide, blinhydroxyurea, idelalisib, olaparib, omacetaxine, palbociclib, pegaspargase, thalidomide, trabectedin
Miscellaneous biological	glatiramer acetate

© 2022 Bajema KL et al. *JAMA Network Open*

Miscellaneous other	leflunomide, sulfasalazine
mTOR kinase inhibitors	everolimus, sirolimus, temsirolimus
Nitrosoureas	carmustine, lomustine, streptozocin
Platinum derivatives	carboplatin, oxaliplatin
Proteasome inhibitors	bortezomib, carfilzomib
Selective adhesion-molecule inhibitors	natalizumab, vedolizumab
T-cell costimulation inhibitors	abatacept, belatacept, alemtuzumab,[b] basiliximab
Thalidomide analogs	lenalidomide, pomalidomide
TNF inhibitors	adalimumab, certolizumab, etanercept, golimumab, infliximab
Topoisomerase inhibitors	irinotecan, mitoxantrone, topotecan
Tubulin-acting agents	eribulin, etoposide, ixabepilone
Tubulin-acting agents (taxanes)	cabazitaxel, docetaxel, nabpaclitaxel, paclitaxel
Tubulin-acting agents (vinca alkaloids)	vinblastine, vincristine, vinorelbine
VEGF tyrosine kinase inhibitors	aflibercept, axitinib, bevacizumab, lenvatinib, pazopanib, ramucirumab, regorafenib, sorafenib, sunitinib, vandetanib

[a]Prescriptions filled within 90 days of positive SARS-CoV-2 test unless otherwise indicated
[b]Filled within one year of COVID-19 diagnosis

© 2022 Bajema KL et al. *JAMA Network Open*

eTable 3. National Institutes of Health (NIH) tiers of prioritization for anti-SARS-CoV-2 therapies[a]

NIH Tier	Risk Group Definition[b]
1	- Receipt of immunosuppressive or cancer medications[c] *or*<br>- HIV with most recent absolute CD4 count ≤2 years is ≤200 cells/mm^3 *or*<br>- Unvaccinated[d] and age ≥75 years *or*<br>- Unvaccinated, age 65-74 years, and ≥1 risk factor for severe COVID-19[e]
2	- Unvaccinated, age 65-74 years, and no risk factors for severe COVID-19 *or*<br>- Unvaccinated, age <65 years, and ≥1 risk factor for severe COVID-19
3	- Vaccinated[d] and age ≥75 years *or*<br>- Vaccinated, age 65-74 years and ≥1 risk factor for severe COVID-19
4	- Vaccinated, age 65-74 years, and no risk factors for severe COVID-19 *or*<br>- Vaccinated, age <65 years, and ≥1 risk factor for severe COVID-19
Other	- Vaccination status not determined (individuals not included in Tier 1)

[a]Per NIH definitions: https://www.covid19treatmentguidelines.nih.gov/overview/prioritization-of-therapeutics/?msclkid=9db67596cf2311ec8b6f159cc2c59087
[b]All factors determined with reference to positive SARS-CoV-2 test
[c]As defined in Supplementary Table 2
[d]As defined in the Supplemental Methods
[e]As defined in Supplementary Table 1

© 2022 Bajema KL et al. *JAMA Network Open*

eTable 4. Factors associated with receipt of different COVID-19 pharmacotherapies among Veterans, January-February 2022[a]

Characteristic	Sotrovimab/ total n/N (%)	Sotrovimab Adjusted OR (95% CI)	Nirmatrelvir/ total n/N (%)	Nirmatrelvir Adjusted OR (95% CI)	Molnupiravir/ total n/N (%)	Molnupiravir Adjusted OR (95% CI)	Remdesivir/ total n/N (%)	Remdesivir Adjusted OR (95% CI)
Age, years								
18-49	80/28,890 (0.3)	0.46 (0.35-0.6)	265/28,890 (0.9)	0.6 (0.51-0.69)	104/28,890 (0.4)	0.45 (0.36-0.57)	58/28,890 (0.2)	0.39 (0.29-0.53)
50-64 (ref)	176/30,749 (0.6)	-	456/30,749 (1.5)	-	239/30,749 (0.8)	-	159/30,749 (0.5)	-
65-74	382/24,815 (1.5)	2.66 (2.22-3.19)	530/24,815 (2.1)	1.42 (1.25-1.61)	300/24,815 (1.2)	1.53 (1.28-1.81)	189/24,815 (0.8)	1.44 (1.17-1.79)
≥75	273/16,133 (1.7)	2.82 (2.32-3.43)	326/16,133 (2.0)	1.29 (1.12-1.5)	214/16,133 (1.3)	1.62 (1.34-1.95)	139/16,133 (0.9)	1.6 (1.26-2.02)
Female sex	73/13,526 (0.5)	1.03 (0.81-1.33)	164/13,526 (1.2)	0.99 (0.84-1.17)	81/13,526 (0.6)	1 (0.79-1.27)	41/13,526 (0.3)	0.77 (0.55-1.06)
Race								
White (ref)	753/74,093 (1.0)	-	1,295/74,093 (1.7)	-	703/74,093 (0.9)	-	428/74,093 (0.6)	-
Black	124/22,756 (0.5)	0.61 (0.5-0.74)	245/22,756 (1.1)	0.63 (0.55-0.72)	135/22,756 (0.6)	0.65 (0.53-0.78)	102/22,756 (0.4)	0.83 (0.66-1.03)
Other	34/3,738 (0.9)	1.13 (0.8-1.59)	37/3,738 (1.0)	0.62 (0.45-0.87)	19/3,738 (0.5)	0.63 (0.4-0.99)	15/3,738 (0.4)	0.83 (0.49-1.39)
Hispanic ethnicity	61/8,609 (0.7)	0.88 (0.67-1.14)	127/8,609 (1.5)	0.97 (0.81-1.17)	47/8,609 (0.5)	0.68 (0.51-0.92)	37/8,609 (0.4)	0.89 (0.64-1.25)
Rural residence	204/16,137 (1.3)	1.31 (1.12-1.55)	298/16,137 (1.8)	1.09 (0.96-1.25)	190/16,137 (1.2)	1.29 (1.1-1.53)	99/16,137 (0.6)	1.08 (0.86-1.35)
Region								
West (ref)	183/23,959 (0.8)	-	322/23,959 (1.3)	-	211/23,959 (0.9)	-	106/23,959 (0.4)	-
Midwest	241/18,831 (1.3)	1.73 (1.41-2.11)	395/18,831 (2.1)	1.55 (1.33-1.81)	196/18,831 (1.0)	1.07 (0.88-1.31)	40/18,831 (0.2)	0.51 (0.35-0.74)
Northeast	191/14,147 (1.4)	1.84 (1.48-2.27)	317/14,147 (2.2)	1.71 (1.46-2.02)	88/14,147 (0.6)	0.66 (0.51-0.86)	136/14,147 (1.0)	2.3 (1.76-3.01)
South	296/43,650 (0.7)	0.99 (0.82-1.2)	543/43,650 (1.2)	0.95 (0.82-1.1)	362/43,650 (0.8)	0.95 (0.8-1.13)	263/43,650 (0.6)	1.47 (1.16-1.88)
COVID-19 vaccination								
None (ref)	245/30,671 (0.8)	-	457/30,671 (1.5)	-	196/30,671 (0.6)	-	147/30,671 (0.5)	-
Partial	61/3,917 (1.6)	1.81 (1.35-2.43)	77/3,917 (2.0)	1.29 (1.01-1.67)	35/3,917 (0.9)	1.3 (0.89-1.9)	36/3,917 (0.9)	1.85 (1.27-2.68)
Full	371/41,418 (0.9)	0.98 (0.83-1.16)	572/41,418 (1.4)	0.87 (0.77-0.99)	336/41,418 (0.8)	1.19 (0.99-1.42)	197/41,418 (0.5)	0.88 (0.71-1.1)
Boosted	234/24,557 (1.0)	0.79 (0.65-0.95)	471/24,557 (1.9)	1.08 (0.94-1.24)	290/24,557 (1.2)	1.47 (1.21-1.78)	165/24,557 (0.7)	0.99 (0.78-1.25)
Smoking								
Never (ref)	327/39,061 (0.8)	-	622/39,061 (1.6)	-	306/39,061 (0.8)	-	207/39,061 (0.5)	-
Former	446/40,274 (1.1)	1.09 (0.94-1.26)	664/40,274 (1.6)	0.92 (0.82-1.03)	380/40,274 (0.9)	1.04 (0.89-1.21)	238/40,274 (0.6)	0.98 (0.81-1.19)
Current	102/17,286 (0.6)	0.78 (0.62-0.97)	236/17,286 (1.4)	0.87 (0.75-1.01)	149/17,286 (0.9)	1.15 (0.94-1.4)	75/17,286 (0.4)	0.87 (0.67-1.14)
Alcohol dependence	137/22,658 (0.6)	0.76 (0.63-0.91)	273/22,658 (1.2)	0.79 (0.69-0.9)	145/22,658 (0.6)	0.78 (0.65-0.94)	91/22,658 (0.4)	0.77 (0.61-0.98)
Substance dependence	23/5,400 (0.4)	0.64 (0.42-0.99)	56/5,400 (1)	0.77 (0.59-1.01)	40/5,400 (0.7)	1.07 (0.77-1.48)	35/5,400 (0.6)	1.31 (0.9-1.91)
Number of underlying conditions								
1-2 (ref)	108/37,502 (0.3)	-	425/37,502 (1.1)	-	167/37,502 (0.4)	-	90/37,502 (0.2)	-
3-4	255/31,289 (0.8)	2.47 (1.95-3.13)	534/31,289 (1.7)	1.38 (1.21-1.58)	277/31,289 (0.9)	1.69 (1.38-2.06)	156/31,289 (0.5)	1.82 (1.38-2.4)
≥5	543/29,852 (1.8)	4.2 (3.34-5.29)	599/29,852 (2.0)	1.41 (1.23-1.63)	410/29,852 (1.4)	2.25 (1.85-2.75)	293/29,852 (1.0)	3.16 (2.42-4.13)

[a]Results of multinomial and binomial models are similar. Multinomial models adjusted for age, sex, race, and ethnicity are presented and limited to Veterans with complete data for all included covariates.

© 2022 Bajema KL et al. *JAMA Network Open*

eTable 5. Factors associated with receipt of any COVID-19 pharmacotherapy among symptomatic Veterans, January-February 2022[a]

Characteristic	Any treatment/total n/N (%)[b]	Any treatment Adjusted OR (95% CI)[c]
Age, years		
18-49	373/15,401 (2.4)	0.48 (0.42-0.54)
50-64 (ref)	768/15,729 (4.9)	-
65-74	1,009/12,221 (8.3)	1.68 (1.53-1.86)
≥75	692/7,581 (9.1)	1.80 (1.62-2.01)
Female sex	273/7,339 (3.7)	0.63 (0.57-0.70)
Race		
White (ref)	2,308/37,204 (6.2)	-
Black	453/11,860 (3.8)	0.63 (0.57-0.70)
Other	81/1,868 (4.3)	0.83 (0.66-1.04)
Hispanic ethnicity	184/4,438 (4.1)	0.78 (0.67-0.91)
Rural residence[a]	575/7,529 (7.6)	1.31 (1.19-1.44)
Region		
West (ref)	606/12,216 (5.0)	-
Midwest	632/9,829 (6.4)	1.29 (1.15-1.45)
Northeast	499/6,175 (8.1)	1.64 (1.45-1.86)
South	1,105/22,712 (4.9)	1.04 (0.94-1.16)
COVID-19 vaccination[b]		
None (ref)	796/15,918 (5.0)	-
Partial	168/2,278 (7.4)	1.38 (1.16-1.65)
Full	1,079/20,998 (5.1)	0.93 (0.85-1.03)
Boosted	799/11,726 (6.8)	1.01 (0.91-1.12)
Smoking[a]		
Never (ref)	1,058/20,025 (5.3)	-
Former	1,250/20,087 (6.2)	1.01 (0.92-1.10)
Current	432/8,914 (4.8)	0.95 (0.84-1.07)
Alcohol dependence[a]	462/1,1437 (4.0)	0.77 (0.69-0.85)
Substance dependence[c]	120/2,971 (4.0)	0.86 (0.71-1.04)
Number of underlying conditions[a]		
1-2 (ref)	560/18,157 (3.1)	-
3-4	870/15,696 (5.5)	1.54 (1.38-1.72)
≥5	1,391/16,195 (8.6)	1.94 (1.74-2.17)
Cancer	823/9,845 (8.4)	1.32 (1.21-1.44)
Cardiovascular disease	1,349/16,376 (8.2)	1.39 (1.28-1.50)
Chronic kidney disease	595/6,688 (8.9)	1.35 (1.23-1.49)
Chronic lung disease	1,171/16,035 (7.3)	1.25 (1.16-1.36)
Diabetes	1,217/15,697 (7.8)	1.35 (1.25-1.46)
Immunosuppressive or cancer medications[d]	392/3,029 (12.9)	2.47 (2.20-2.78)
Mental health conditions[e]	1,115/22,342 (5.0)	1.05 (0.97-1.14)

© 2022 Bajema KL et al. *JAMA Network Open*

Obese (body mass index ≥30 kg/m^2)	1,466/26,363 (5.6)	1.16 (1.08-1.26)

[a]Includes sotrovimab, nirmatrelvir, molnupiravir, and remdesivir. Symptomatic Veterans had any of 15 pre-specified COVID-19-related symptoms present on the day of positive SARS-CoV-2 test or within the preceding 30 days.

[b]A total of 3,079 Veterans who received any COVID-19 pharmacotherapy out of 56,206 Veterans testing positive for SARS-CoV-2 were included. Models were limited to Veterans with complete data for all included covariates.

[c]All models adjusted for age, sex, race, and ethnicity.

© 2022 Bajema KL et al. *JAMA Network Open*

eReferences

1. Centers for Disease Control and Prevention. COVID-19 Vaccines for Moderately of Severely Immunocompromised People. Accessed June 3, 2022. https://www.cdc.gov/coronavirus/2019-ncov/vaccines/recommendations/immuno.html

© 2022 Bajema KL et al. *JAMA Network Open*
